# Gender Diversity on Boards of Directors and Remuneration Committees: The Influence on Listed Companies in Spain

**DOI:** 10.3389/fpsyg.2018.01351

**Published:** 2018-08-17

**Authors:** Antonio L. García-Izquierdo, Carlos Fernández-Méndez, Rubén Arrondo-García

**Affiliations:** ^1^Department of Psychology, Universidad de Oviedo, Oviedo, Spain; ^2^Department of Business Administration, Universidad de Oviedo, Oviedo, Spain

**Keywords:** remuneration, gender-discrimination, minority influence, board of directors, remuneration committee, management, listed companies

## Abstract

Women have traditionally been underrepresented on boards of companies, but after some social and legal pressure their presence has been increased during recent years. This paper examines the relation of the presence of female directors both at board meetings and at audit and remuneration committees, with CEO pay and the shareholders' consultative vote on managerial remuneration plans (“say on pay”). Using a large sample of Spanish firms listed between 2011 and 2015, our study reveals that firms with female representation on their remuneration committee, display lower levels of CEO pay and CEO pay growth. We also obtain evidence that this effect is attributable to the proprietary female directors. Additionally, from the “say on pay” perspective, female membership of the remuneration committee is associated with a lower number of votes in terms of director remuneration reports and related policies. Overall, our results indicate that female directors on the remuneration committee contribute to a moderation of executive remuneration growth and are consequently perceived by shareholders as valuable resources in the design of executive remuneration plans. This confirms the influence of the minority group, females, in the sustainable progress of these companies. Our results support the presence of female directors not only as a social measure or tokenism, but also as a contribution to good governance practice.

## Introduction

Cultural, gender and racial composition of boards of directors are counted amongst the most relevant concerns for managers, shareholders, and directors of major corporations (Carter et al., 2003). In the Strategic Engagement for Gender Equality 2016–2019, the European Commission maintain their commitment to improve the gender balance in economic leadership positions, in particular obtaining at least a 40% representation of the sex that is currently less represented within the group of non-executive directors in listed companies (European Commission, 2016-2019). The mandate of the European Commission urging EU member States to increase the presence of female directors has resulted in legislative reforms favoring their progressive incorporation to the boards of listed companies. The incorporation of female directors to the firms' boards is aimed at ensuring equality in decision-making, but it could also be justified on the grounds of their specific ability to improve company decision-making.

The increase of female presence on corporate boards has been promoted either by imposing legal quotas or by issuing softer recommendations with respect to the codes of good governance. Scandinavian countries have been pioneers in the use of a quota system. In 2003 the Norwegian parliament passed a law requiring 40 per cent of corporate directors in listed companies to be women (Rose, 2007). Other countries such as Belgium, France, Germany, Iceland, India, Israel, Italy, and Spain use some forms of quota system to improve female representation on the boards of listed firms. Nonetheless, the quota system has been a matter of discussion due to their convenience and acceptability when dealing with meritocracy (e.g., Moscoso et al., 2012), as it could be seen as a tokenism and/or as granting less value. However, based on historical disadvantages and to offer an opportunity to “rebalance” the society, the Spanish Organic Law 3/2007 on effective equality between men and women has granted large companies 8 years to include a minimum proportion of 40% female directors on their boards. Nevertheless, this level of presence on the part of female directors has not been achieved so far.

In the case of Spain, the legal system of quotas co-exists with the softer recommendations proposed by the code of good governance which establishes that in 2020, female directors should account for 30% of the board seats of listed companies. The last report issued by the Spanish Association of Women Entrepreneurs (Women CEO, 2017), reveals during the last year an increase in the percentage of female directors at the IBEX 35 companies from 19.1 to 21.8% and that for the first time, all firms in this index have female representation in their boards.

The under representation of female directors on the boards is not only a Spanish issue, but is common to most OECD countries, also. Although there has been a steady increase in the number of female directors over recent years, the OECD (2017) report on the implementation of the OECD gender recommendations shows that female directors constitute still a minoritarian group in the boards of listed companies. In 2016 female directors accounted for only 20% of the directorships in listed OECD companies, and as few as 4.8% of chief executive officer (CEO) positions were occupied by women. These figures were slightly higher than the averages of 16.4 and 2.4% for female directors and CEOs respectively in 2013.

Although female directors represent a minority within the boards of listed companies in developed countries, the presence of minorities in boards of directors can influence decision-making. In this sense, even in male dominated boards, appointing female directors may improve the working environment of the group (Bilimoria and Huse, 1997), and as a result, gender diverse boards meet more frequently and suffer less from absenteeism (Adams and Ferreira, 2009). There is a growing body of studies that analyse the effect of gender diverse boards on firms' affairs. The results obtained are not conclusive so far. There is some evidence suggesting that female directors improve firms' performance (Adams et al., 2011; Hutchinson et al., 2015; Terjesen et al., 2016) and also that it has a negative impact on the firm's stock prices (Ahern and Dittmar, 2012). The effect of female directors on firm's risk is also unclear with some studies suggesting a reduction of firms' risk taking (Lenard et al., 2014; Liu et al., 2014; Hutchinson et al., 2015) and others find no effect at all (Sila et al., 2016). However, the identification of a clear relationship between boards' gender diversity and firms' metrics of risk might be impeded by the fact that risk measures such as stock volatility or its systematic or idiosyncratic components are subject to many external factors uncontrolled by the board.

Our study follows a more direct approach to analyse the female's influence on the boards' functioning. Instead of focusing our attention on metrics of firms' risk, we analyse the association between female directors and a board decision with a high impact on firm risk taking: managerial pay. A question of special interest in the company is the way in which the board of directors uses compensation systems to align interests between shareholders and managers. From this perspective, we study the role played by female directors on managerial remuneration design as a determinant of the company's risk level.

The board of directors and its remuneration committee are both responsible for hiring, firing, and designing the compensation of top managers. Setting top managerial remuneration is one of the most problematic decisions at firm level, since top managers can exert a major influence on the bodies responsible for this decision (i.e., the board and its remuneration committee). Powerful managers can tilt the balance in favor of their own interests when deciding managerial pay levels and structure. Managerial power can often be behind the growth in executive remuneration and is more often than not unjustified by firm growth or profitability. In order to avoid managerial risk aversion, the remuneration packages of top managers have incorporated variable elements based on firms' performance as well as equity and option based pay. The positive relation between the value or stock options and the firms' stock volatility constitutes a source of risk-taking incentives for top managers. An inadequate pay mix can greatly alter managerial attitudes toward risk taking, resulting in an incentive for misalignment. An excessive use of options-based compensation has been attributed as being one of the main causes of the global financial crisis in 2007 (Fahlenbrach and Stulz, 2011; Chesney et al., 2012; Gregg et al., 2012; Cheng et al., 2015). Similarly, there is evidence of a positive association between the use of options-based CEO remuneration and socially irresponsible activities (McGuire et al., 2003; Bouslah et al., 2018).

Our contribution to the literature on board diversity is twofold. First, by focusing on the analysis of female directors and how the latter can influence the direct outcome of boards' decisions (i.e., CEO pay). Rather than analyzing a firm level outcome such as firm risk, we try to isolate our results from the confounding effects of outside factors that may affect firms' risk or performance. Second, we contribute to the scant group of works analyzing the effect of board gender diversity on CEO pay setting. To our knowledge, the only evidence provided by Adams and Ferreira (2009) uses a sample of large US firms. Our analysis has been conducted for the Spanish market. Therefore, the risk reducing influence of female directors observed in the US market might differ in the case of Spain where the use of option-based managerial compensation is much less common than in the US context. Our results could be extrapolated to other European countries with similar CEO pay structures. This is indeed a matter of interest given that Union member countries are expected to implement UE directives seeking board gender diversity.

The remainder sections of the paper are structured as follows. In section Objective we do a review of previous literature. In the next two sections we describe our data and empirical approach. We follow by presenting and discussing our results in section Results and finally in section Discussion we extract conclusions.

## Gender diversity, discrimination, and management

### Female under representation at the upper echelons

Women have traditionally been excluded from the active work force and remain under-represented in managerial positions (Tatli et al., 2013), experiencing the “glass ceiling” effect (Pichler et al., 2008; Cech and Blair-Loy, 2010), and consequently remain in an unfavorable situation. According to the 2010–2016 national labor force survey conducted by the Pew Research Center, women corporate power is limited although women represent 40% or more of the work force in more than 80 countries globally. Additionally, the total number of female directors and the rate of increase in their numbers over time remain somewhat scarce. Nevertheless, women have been gaining ground on corporate boards in recent years. In 2015, women held 17.9% of the board seats in Fortune 1,000 companies. The proportion of female directors on the boards of large public firms listed in EU 28 countries has increased from 18.6% in 2014 to 23.3% in 2016. Female board members in IBEX35 firms increased by 13.75% in 2016 and the total percentage of female directors in the IBEX-35−19.83%—is slowly getting closer to the European average. These meager figures of female representation show that there is still a long way to achieving equal career opportunities for men and women.

Legislation has encouraged diversity in management teams, but both intentional and unintentional institutional barriers exist in the workplace (Joshi et al., 2015). Even when they are in high status occupations, women often face challenges at work, including discrimination (Hahm et al., 2010). As a result, they experience issues of visibility, that is, increased experiences of heightened visibility and invisibility (Broadbridge and Simpson, 2011).

We can trace the roots of female discrimination at work from two main but different perspectives: individual and organizational. The organizational perspective would seem to be more relevant in this case, with some approaches dealing with similar problems, mainly gender stereotyping of managerial positions (Schein, 2007), so we are going to outline the organizational perspective. Stone-Romero and Stone (2005) “presented an interesting model that analyses discrimination” from the perspective of decision-makers. It highlights discrimination as being the result of categorizing individuals according to group membership in a way that decision-making is influenced by stereotypes. In general, women stereotypes are “viewed as less suitable for managerial jobs when compared to the stereotypes of male, white, middle-aged workers (Goldman et al., 2006),” who seem to represent hard or technical competences (i.e., knowledge, abilities, self-confidence, assertiveness, and dominance). The fact that these demands are key to accessing high-status managerial and professional jobs contributes to the problem; even more so, when women are associated with female soft competences (i.e., teamworking, communication, and emotional support). Therefore, criteria for job access are presumably fostered and biased because of this kind of stereotyping (Schein, 2007).

The social identity perspective (Tajfel and Turner, 1979), “is mainly based on stating” that group members (ingroup) tend to protect their self-esteem in order to achieve a positive and particular social identity. This can result in discrimination, either directly or indirectly, competing, separating or punishing the outgroup by giving preferential treatment to the ingroup, and ultimately, leading to the so-called ingroup bias phenomenon. In this sense, the statistical discrimination model deals with the stereotypical orientation of the employer on average data about the possible performance of a particular group, and protecting the ingroup as well. According to this theory, inequality may exist and persist between demographic groups even when economic agents are non-prejudiced. Following Rodgers (2009), this type of discrimination can result in a self-reinforcing vicious circle over time, as on exit, average individuals from the discriminated group are discouraged from participating in the market or improving their skills as their (average) return on investment is less than for the non-discriminated group. But there are other approaches for the explanation of gender discrimination.

The occupational segregation model refers to the case where the representatives of different groups of workers have unequal access to employment positions. This means that occupations are separated: some are for men and others for women. The origin of segregation can occur on different grounds. To begin with, self-selection phenomenon, the selection by women to depart from higher level positions choosing instead to dedicate themselves to more traditional family roles. The latter leads us to the glass escalator effect, through which women must watch as men surpass them on the way to the top of the organizations (Snyder and Green, 2008).

Sheridan and Milgate (2003) “attribute the lack of female directors to the scarce number of women as senior managers.” The case of management is particularly interesting since it has traditionally been viewed as a male domain requiring specific sets of skills. But simultaneously, women who are on the way to getting power are at risk for the backlash effect (Rudman, 1998), seen as a social and economic punishment for defying stereotypic expectations, and consequently at risk of prejudice and hiring discrimination. Working women who demonstrate stereotypical male behaviors are likely to face setbacks because they do not fit the female stereotype, especially in top positions. One explanation of this phenomenon is that women who demonstrate hard skills that are consistent with successful managers, can be perceived negatively by some co-workers for not behaving in a traditional feminine way. These women are perceived as more competent, but at the same time less socially skilled, less likable and less likely to be promoted.

Moreover, women need to comply with masculine norms of behavior if they are to break through the glass ceiling, acting as masculine manager stereotypes, and exceeding male cultural norms. Liff and Ward (2001, p. 20) pointed out that “organizations are also the site within which women come to understand the requirements of senior jobs and their own career options. Organizational cultures, structures, and practices provide the context within which this occurs and can lead women to decide that such jobs, or the process they would have to go through to get them, are not for them.” This can lead to a generalized managerial assumption that women are satisfied to continue in their present position, whilst male peers indicate much more strongly to the person responsible for promotions their readiness for the next step up. Traditional female family responsibilities could hinder commitment to the organization and their lack of involvement in corporate networks limits the access of women to senior jobs. The gender bias seems to rest on employers' stereotypes of women, as more trustworthy, honest, meticulous and patient (Aganon, 1999).

Other explanations present women as more skilful than men in terms of team-building and communication but worse in terms of business skills. Based on these attitudes, women and men are therefore matched to different jobs, “which require different traits, consistent with the views of the dominant group holding that job.” Outstanding research by Schein and colleagues over a period of more than 40 years showed that the role of “managers has been associated with male characteristics, not only in the United States” but in several other countries as well (e.g., Schein, 2007), posing the think manager-think male effect. This situation might explain by itself that white men hold a disproportionate number of the highest paying jobs and account for the best opportunities for advancement. Therefore, white men will be less disadvantaged at this stage than white women. Managers and employees of professional firms tend toward the homosocial reproduction in organizations where they were drawn, hiring co-workers similar to themselves.

Following Goldman et al. (2006), the consequences of gender discrimination can exist both at an organizational level and at an individual level also. For example, discrimination affects negatively the psychological health of women, and even stigmatizes them if they publicly denounce having been discriminated.

Summing up this section, women have been suffering the personal and social consequences of a disadvantageous situation both outside and inside organizations due to several cultural and biased decision-making practices revolving around discrimination issues, mainly based on stereotyping their competences in order to avoid them holding managerial positions. But, as we can see in the next sections the contribution of women to organizational performance should be highlighted as their role could be beneficial for organizations in many ways. Now we focus on the influence that female directors can have on firm value in the form of financial performance and risk-taking with specific attention being paid to their role in executive pay setting.

### Female directors and boards performance

The Board of Directors is a control governance mechanism, aimed to monitor managerial activities so as to mitigate agency costs (Jensen, 1993), and to set the strategic objectives which should orientate the course of the company (Hillman and Dalziel, 2003). The Board's supervisory tasks include: monitoring the CEO, and the implementation of the firms long term strategy, firing and hiring the CEO and assessing and rewarding the CEO/top managers of the firm (Hillman and Dalziel, 2003).

There are opposing views on the forces driving executive remuneration design. According to the optimal contracting perspective on executive remuneration, managerial remuneration packages should align the divergent interests of managers and shareholders. In this regard, remuneration constitutes a mechanism which provides incentives and discipline to executives, ensuring that the marginal benefits that can be obtained from opportunistic behavior are lower than the opportunity costs associated with this behavior in terms of lower payments from the company (Shleifer and Vishny, 1997).

In contrast, the managerial power approach (Jensen, 1993; Hermalin and Weisbach, 1998; Bebchuk et al., 2002a,b; Bebchuk and Fried, 2003) argues that the design and implementation of executive remuneration policies is a potential source of conflict as powerful executives can influence managerial compensation design, which can result in sub-optimal compensation contracts. An executive remuneration structure should be aligned with the interests of shareholders and other employees, company's performance, long-term strategy and corporate and social responsibility commitments. Therefore, regulators and corporate good governance codes propose, among other recommendations, that the board of directors is dominated by independent directors and hence, set up a nomination and remuneration committee (hereafter, NRC) to independently evaluate the remuneration policy of company executives and oversee that it is performance-related, and complies with the principles of moderation and transparency.

The effective implementation of the board of directors' functions involves designing an adequate system of incentives for executives and, at the same time, ensuring that the board structure is diverse in terms of gender, races and experience of their members (Westphal and Milton, 2000). The Higgs Report (2003) suggested that gender diversity increases board effectiveness and therefore recommended that more women be incorporated to boards, which has long been considered an organizational good practice. The arguments for gender diversity on corporate boards are manifold. Gender diverse boards provide access to a broader knowledge base (Sheridan et al., 2011; Laguir and Den Besten, 2016), which in turn expands the set of experiences and points of view available in an all-male board (Daily and Dalton, 2003). As a work group, adding female directors to the boards' composition improves the working environment (Bilimoria and Huse, 1997), reduces directors' absenteeism (Adams and Ferreira, 2009) and produces better board deliberations (Eagly and Johnson, 1990; Kravitz, 2003). Having female representation on the board also has the advantage of adding legitimacy to the firm, as inequality between men and women's rights is seen as unacceptable in developed countries. From the agency theory perspective gender diverse boards are also optimal, since they produce superior monitoring outcomes as compared to all male boards (Adams and Ferreira, 2009). There are also numerous studies that have linked gender-diverse boards with better financial performance (Carter et al., 2003; Erhardt et al., 2003; Smith et al., 2006; Campbell and Mínguez-Vera, 2008; Lückerath-Rovers, 2013; Liu et al., 2014; Terjesen et al., 2016), better governance (Adams and Ferreira, 2009; Gul et al., 2013), increased innovation (Miller and Triana, 2009; Torchia et al., 2011), and firms' corporate social responsibility (Bear et al., 2010). Nonetheless, as female are a minority group, it is not clear how their influence could take place. Following Minority Conversion Model (Moscovici, 1985), minorities activate social validation process focusing on the object of disagreement. That is, minorities influence take place indirectly through conversion, a latent change, intimate; whilst majorities influence take place through a direct way, fast and in public (Mugny et al., 1991). Social comparison operates more on different opinions (Nemeth, 1986). When we compare majority groups with minorities some social differences emerge (Maass, 1991): minorities are seen as more salient, different, with less credibility, and being under high social pressure. Following excellent Mannix and Neale (2005) revision on this topic we are going to address deeply this issue. Majority groups has been traditionally consider as more powerful and influencers through the pressure for conformity (e.g., Janis, 1982), because it is assumed integration and social validation. However, Westphal and Milton (2000) demonstrated that the influence of minorities can avoid outgroup biases that would otherwise minimize their influence when they have prior experience on other boards or social network links to other directors that foster them to create the image of similarity with the majority. As Nemeth (1986) stated, “minority opinions provoke majority members to respond with an augmented cognitive flexibility, probably because of the social pressure of the team to converge to a single decision or consensus” (Moscovici, 1985), and in the case of boards presenting a documented remuneration proposal. Mugny and Papastamou (1980) found that “the consistent disagreement of two is stronger than one disagreement.” More on this Larson et al. (2004) found coalitions to be particularly effective at persuading the group. Finally, Moscovici (1985), proposed that double minorities (different in demographic characteristics and opinions) might exert greater latent influence than in-group minorities (similar to the majority in their demographic characteristics but have divergent opinions). Outgroup members with divergent perspectives may be more willing to express opinions and exert influence on the group, avoiding cognitive dissonance and enhancing the out-group member to validate her contribution to the group.

There is also a large body of literature that investigates the effect of board gender diversity on firm risk. The study of Lenard et al. (2014) shows that more gender diversity in a board of directors impacts firm risk by contributing to lower variability of stock market return. Results in Australia by Hutchinson et al. (2015) also show that gender diversity moderates excessive firm risk which in turn improves firms' financial performance. Faccio et al. (2016), found that transitions from male to female CEOs (or from female to male) are associated with economically and statistically significant reductions (increases) in corporate risk-taking. On the contrary, Sila et al. (2016), have not found evidence that female board representation affects firm risk in a sample of US firms. Croson and Gneezy (2009) found that women could be more risk averse in the general population but not in managerial positions where differences are smaller and often inexistent. Here, a self-selection phenomenon could be at the origin of this exception. Another explanation can be grounded on the situational theory by Mischel (1968, 2004), who established that when the situation is strong enough (e.g., limited with social norms, rules, etc.), personality differences between subjects are less explicit. In consequence, behavior is more specific and unstable depending on the situation, in a way that when the latter is unambiguous behavior is more similar.

### Female directors and executive pay

In order to avoid possible conflicts of interest interfering in the optimal design of remuneration policies, the board and NRC need a high degree of independence, experience, knowledge, expertise, and values (Hillman and Dalziel, 2003). In this sense, gender diversity can help provide these skills, necessary for the optimal design of remuneration policies. Several prior studies have examined the effect of gender diversity on NRC when designing executive pay arrangements. This scarce but emerging literature investigates mainly whether top management pay and corporate performance are more aligned in companies with gender-diverse compensation committees, leading to mixed results. The study by Borrenbergs et al. (2017), investigates the relationship between female presence in the remuneration committee and the relative weight of performance contingent pay for top executives. The findings, based on a detailed analysis of a sample of public US and Canadian firms, show strong evidence supporting the fact that gender-diverse compensation committees are associated with lower levels of annual bonuses in top executives' remuneration contracts (variable short-term compensation). However, the models do not provide evidence with respect to the relative weight of total variable compensation, that is, when other long-term variable compensations are considered (option awards, stock grants and long-term incentive plans). Other studies have found evidence of a negative relationship between gender diversity compensation committees and CEO pay. A study by Bugeja et al. (2016), based on annual company data collected between 2002 and 2009 show that CEO compensation levels are negatively associated with the gender-diversity of the compensation committee, but not with the gender-diversity of the board.

## Objective

In this study we have focused on the role played by female directors on boards and remuneration committees when designing and implementing CEOs incentive schemes. Our work expands upon previous research on the association between gender-diverse boards (and compensation committees) and CEO remuneration packages by analyzing whether the percentage of dissent say on pay vote is different for firms with and without gender-diverse compensation committees.

Taking into account all of the above considerations regarding the positive association of female directors with board monitoring performance, we can hypothesize that the presence of female board members is positively associated with CEO wage moderation. Consequently, we can hypothesize the influence of females as minority group on male majority group on boards.

Also, when considering the negative association of female directors with firm risk and risk-taking incentives provided by option-based remuneration, we expect to see a negative association between female director representation and the use of this form of incentive pay.

## Methodology

### Sample and data

Our sample includes unbalanced panel data from companies listed on the Madrid Stock Exchange between 2011 and 2015. Companies going public between 2011 and 2015 have been added to the sample in the year of their initial public offer and companies de-listed have been eliminated in the year of that event. We have obtained all corporate governance information related to board structure, committees, CEO remuneration and ownership structure of the firms from the registers of the National commission of the stock Exchange (Comisión Nacional del Mercado de Valores or CNMV). The accounting data to determine the size of the company, its profitability and leverage comes from the records of the CNMV and SABI (Sistema de Análisis de Balances Ibéricos) databases. Finally, the share prices to estimate market returns are obtained from Capital IQ.

### Variables

The main variables of our study are CEO pay and the results from the annual vote issued by the firms' shareholders about the firm's executive compensation plan, these being used as dependent variables. As we are mainly interested in the moderation and risk incentives provided by executive pay, we analyse the relative annual increase of CEO pay and the proportion of equity and option-based CEO pay. The first is proxied by the annual increase of total CEO pay scaled by the previous CEO total pay. The second is proxied by the ratio of equity and option-based CEO pay components scaled by total CEO pay.

Our main independent variables are the presence of female directors on the board and the nomination and remuneration committee. We proxy the female influence on the firm's corporate governance system with three variables, that is, by the proportion of female directors scaled respectively by the number of members on the board, the audit committee, and the nomination and remuneration committee. Alternatively, we use a set of dummy variables that take the value of one when there is at least one female director on the board, the audit committee, or the nomination and remuneration committee. The influence of female directors on executive compensation may differ depending on the independent status of the directors considered. Therefore, to investigate the possible differences between executive, independent and proprietary female board members on executive pay, we have included the proportion of executive independent and proprietary female directors in relation to the total board size. We also include in our regression models the corresponding dummy variables representing the presence of such directors on the board.

We control for both board and committee composition and size that are considered to affect their supervisory activity. We use the proportion of independent board directors as a proxy of the board's independency. The size of the board and its committees are controlled by the logarithm of the total number of board directors.

We control possible firm size effects on all models through the inclusion of the logarithm of the book value of total assets measured in thousands of constant Euros. We also include controls for financial leverage and performance and investment opportunities that may affect CEO pay. We proxy the firm's performance with two different variables: company Return on assets, measured by the ratio of EBIT to total assets and the market annual performance. Finally, we control for the firm's investment opportunities by including the market to book ratio defined as the market value of the firm^‵^s equity scaled by its book value.

The models account for the possible effects of the changes in general economic conditions by including year dummies. As executive pay may vary across industries, we have also included Standard Industrial classification (SIC) dummy variables to control for this effect.

### Data analysis

The following regression equation is used to test our hypotheses related to two alternative outcomes: the annual growth rate of CEO pay and the proportion equity and option-based CEO remuneration;

(1) $$\begin{array}{l} y_{i,t}=\alpha +\beta^{\prime }{\text{M}}_{i,t}+\delta^{\prime }{\text{X}}_{i,t}+\mu_{i}+\lambda_{t}+\varepsilon_{i,t} \end{array}$$

where our dependent variable, *y* indexes either a CEO pay growth rate or the proportion of equity and option-based CEO pay by firm *i* in year *t*, M is a vector of variables of interest that are potential determinants of CEO remuneration—female representation on the board, the remuneration committee and the audit committee. X is a control vector that includes board independence and size, firm size, profitability, leverage, and investment opportunities variables. Our model also includes industry (μ_*i*_) and year (λ_*t*_) fixed-effects and allows for heteroscedastic error terms that are clustered at firm level (ε_*i, t*_).

## Results

Table 1 shows the distribution of the sample. Gender-diverse board of director companies (i.e., boards that have at least one female director) have increased from 59.24% in 2012 to 75.54% in 2015. We can attribute the rise of female participation on the board of directors to the progressive implementation of the Spanish Organic Law 3/2007, regarding effective equality between men and women. This Law prescribes large listed companies to reach a 40% female representation on the board of directors in 8 years.

Table 1Sample description.

**Table:** 

**(A) Frequency of firms by year (2012–2015)**.					
	**Total**	**Female presence on boards**		**All-male board of directors**	
**Fiscal year**	**Number firms**	**Number firms**	**Percentage**	**Number of firms**	**Percentage**
2012	157	93	24.16%	64	30.34%
2013	153	87	22.60%	66	31.28%
2014	147	100	25.97%	47	22.27%
2015	139	105	27.27%	34	16.11%
Total	596	385	100%	211	100%

**Table:** 

**(B) Frequency of firms by year: all-male vs. female presence on boards**.					
	**2012**		**2013**		**2014**		**2015**	
**Type of board**	**Number firms**	**Percentage**	**Number firms**	**Percentage**	**Number firms**	**Percentage**	**Number firms**	**Percentage**
All-male	64	40.76%	66	43.14%	47	31.97%	34	24.46%
Gender-diverse	93	59.24%	87	56.86%	100	68.03%	105	75.54%
Total	157	100%	153	100%	147	100%	139	100%

The descriptive statistics of the variables used are presented in Table 2. The mean annual total CEO remuneration is 1,487,769 Euros with an average annual increase of 6.72% over the period 2012–2015. We have reported an average 2.83% of dissent says on pay vote, resulting in a much lower opposition to executive remuneration plan than in the US market. For instance, in the same period of our study, the consultancy firm Semler Brossy reports for the Rusell 3,000 firms that the opposition say on pay vote ranged from a minimum level of 8% in 2016 to a maximum level of 10% in 2012. The proportion of female directors is 11% for the board and the remuneration committee and 13% for the audit committee. This 11% proportion of female directors is comprised of independent directors (5.3%), proprietary directors (4.9%) and executive directors (0.7%). To conserve space, we omit discussion of the descriptive statistics of our control variables.

**Table 2:** Descriptive statistics.

**Variable**	**Number of Observations**	**Mean**	**Standard Deviation**	**Minimum**	**Maximum**
Annual CEO pay (thousand euros)	243	1,487.769	29,336.24	31	241,919
Relative annual increase of CEO pay	243	0.067	0.9376	−0.966	6.2711
Proportion of dissent say on may vote	329	0.028	0.0758	0	0.0933
Proportion of board female directors	243	0.115	0.1036	0	0.5
Proportion of audit committee female directors	236	0.132	0.1730	0	0.6666
Proportion of remuneration committee female directors	213	0.112	0.1720	0	0.8
Proportion of proprietary board female directors	243	0.05	0.0775	0	0.3846
Proportion of executive board female directors	243	0.007	0.0310	0	0.2222
Proportion of independent board female directors	243	0.058	0.0797	0	0.5
Female directors presence (board)	243	0.712	0.4537	0	1
Proprietary female directors presence (board)	243	0.387	0.4880	0	1
Executive female directors presence (board)	243	0.058	0.2334	0	1
Independent female directors presence (board)	243	0.399	0.4907	0	1
Proportion of equity and option based CEO pay	243	0.103	0.3648	0	2.7663
Proportion of board independent directors	243	0.335	0.1747	0	0.8888
Board size	243	11.49	4.717	1	36
Remuneration committee size	213	4.333	1.7174	1	12
Audit committee size	243	4.097	1.5419	1	12
Total assets (thousand euros)	243	3,596,727	7,753,106	12,039.73	5.01E + 07
Return on assets	243	0.035	0.1213	−0.428	0.3614
Annual market return	243	0.1	0.4898	−0.823	2.0217
Book to market ratio	243	1.884	2.7633	−1.138	22.129

Table 3 shows the univariate analysis of the mean differences between companies with and without female directors (gender-diverse and all-male board of directors) for the period 2012–2015. Companies with female directors offer a higher CEO total remuneration, a higher percentage of short-term and long-term variable remuneration than those companies with all male boards. The mean value of CEO total pay is 461.83 thousand euros higher for firms with gender-diverse boards of directors. We also note that companies with gender diverse board directors are bigger and show both a higher percentage of independent directors and larger Boards.

**Table 3:** Comparisons of firms with and without female directors between 2012 and 2015.

**Variable**	**Firms with female directors**	**Firms with no female directors**	**Mean difference**	**t**
	**Mean**	**Mean**		
Annual CEO pay thousand euros	1342.64	880.80	461.83^***^	26.472
Relative annual increase of CEO pay	0.0278	0.08113	0.0532	0.4549
Proportion of equity and option based CEO pay	0.3364	0.0616	0.0537	1.2738
Proportion of dissent say on may vote	0.0285	0.0189	0.0096	14.639
Proportion of board independent directors	0.3364	0.2855	0.0508^***^	53.644
Board size	11.0727	9.3044	1.7683^***^	84.843
Logarithm of total assets	12.5226	12.4360	0.0866	0.6282
Return on assets	0.0246	0.0211	0.0035	0.3129
Annual market return	0.0687	0.0875	−0.0187	−0.6226
Book to market ratio	2.6056	3.1693	−0.5636	−0.9521
Debt to assets ratio	0.5927	0.6839	−0.0911	−11.117

*** *p < 0.01. In bold statistically significant coefficients of variables of interest*.

Tables 4–**7** show our estimations of the effect of female directors both on the growth rate of CEOs' pay and the proportion equity and option-based CEO remuneration. **Table 8** displays the effect of female directors on the percentage of shareholders opposition to the managerial remuneration plan presented at the Annual General Meeting (negative or dissent say on pay vote). Results in Tables 4, 5 show respectively that the proportion of female directors on the remuneration and nomination committee and the indicator of the presence of female members on this committee both relate negatively to the growth rate of CEO pay. The presence of female directors on the board committee specifically responsible for the design of executive pay design is associated to a moderation in the growth of CEO pay.

**Table 4:** Regression results of CEO pay on female directors' weighting.

**Variable**	**Relative annual increase of CEO pay**						**Proportion of equity and option based CEO pay**					
	**Model 1**		**Model 2**		**Model 3**		**Model 4**		**Model 5**		**Model 6**	
	**B**	**t**	**B**	**t**	**B**	**t**	**B**	**t**	***B***	**t**	**B**	**t**
Proportion of board female directors	−0.271	−0.51					0.108	0.58				
Proportion of audit committee female directors			0.477	1.39					0.0622	0.50		
Proportion of remuneration committee female directors					−0.761^**^	−**2.43**					−0.0350	−0.30
Proportion of board independent directors	0.264	1.13	0.231	1.04	0.278	1.24	−0.176	−1.64	−0.176	−1.58	−0.240^**^	−2.11
Logarithm of board size	0.119	1.08	0.187	1.28	0.184	1.11	0.0262	1.01	0.0229	0.76	0.0396	1.36
Logarithm of total assets	−0.0135	−0.48	−0.0163	−0.53	−0.0225	−0.69	0.000935	0.16	0.000735	0.13	0.00157	0.25
Return on assets	−0.350	−0.96	−0.332	−0.91	−0.0130	−0.04	−0.00798	−0.09	−0.0126	−0.15	−0.00624	−0.07
Debt to assets ratio	−0.0932	−0.62	−0.0610	−0.41	−0.190	−0.97	0.0124	0.48	0.0144	0.50	0.0101	0.28
Annual market return	0.0634	0.69	0.0225	0.25	0.106	1.00	−0.0542^*^	−1.91	−0.0525^*^	−1.85	−0.0530^*^	−1.73
Book to market ratio	0.0151	1.55	0.0160^*^	1.74	0.00505	0.74	−0.000805	−0.36	−0.000562	−0.25	0.000343	0.15
Constant	0.137	0.31	−0.113	−0.26	0.217	0.43	−0.0518	−0.40	−0.0374	−0.30	−0.0528	−0.42
*No. of Obs*.	243		236		213		297		290		264	
*Adjusted R^2^*	0.0587		0.0712		0.0597		0.180		0.178		0.157	
*F*	4.927		4.446		3.864		1.788		1.767		1.426	
*p*	0.0000		0.0000		0.0000		0.0409		0.0441		0.145	
*No. of firms clusters*	92		92		84		95		95		87	

Regressions are estimated using OLS with standard errors clustered by company. All models include 2-digit SIC and year dummies.

* p < 0.1;

** *p < 0.05. In bold statistically significant coefficients of variables of interest*.

**Table 5:** Regression results of CEO pay on female directors' presence.

**Variable**	**Relative annual increase of CEO pay**						**Proportion of equity and option based CEO pay**					
	**Model 1**		**Model 2**		**Model 3**		**Model 4**		**Model 5**		**Model 6**	
	**B**	**t**	**B**	**t**	**B**	**t**	**B**	**t**	**B**	**t**	**B**	**t**
Female directors presence at the board	−0.0501	−0.43					0.0561	1.15				
Female directors presence at the board			0.176	1.48					0.0165	0.38		
Female directors presence at the board					−0.232^**^	−**2.01**					0.00693	0.13
Proportion of board independent directors	0.258	1.19	0.213	0.97	0.313	1.29	−0.182^*^	−1.66	−0.177	−1.59	−0.247^**^	−2.02
Logarithm of board size	0.128	1.08	0.180	1.25	0.166	1.01	0.0138	0.63	0.0216	0.73	0.0412	1.33
Logarithm of total assets	−0.0139	−0.49	−0.0145	−0.48	−0.0248	−0.75	0.00227	0.35	0.00101	0.17	0.00128	0.21
Return on assets	−0.344	−0.91	−0.346	−0.95	0.0173	0.05	−0.00718	−0.08	−0.0136	−0.16	−0.0173	−0.17
Debt to assets ratio	0.0597	0.64	0.0197	0.22	0.102	0.99	−0.0544^*^	−1.93	−0.0523^*^	−1.82	−0.0534^*^	−1.74
Annual market return	0.0152	1.54	0.0156^*^	1.69	0.00582	0.90	−0.00114	−0.51	−0.000642	−0.28	0.000292	0.13
Book to market ratio	−0.0934	−0.61	−0.0678	−0.47	−0.191	−0.96	0.0140	0.52	0.0127	0.46	0.0112	0.31
Constant	0.126	0.28	−0.111	−0.25	0.270	0.53	−0.0703	−0.52	−0.0341	−0.27	−0.0581	−0.43
*No. of Obs*.	243		236		213		297		290		264	
*Adjusted R^2^*	0.0584		0.0720		0.0545		0.184		0.178		0.157	
*F*	4.862		4.492		3.818		1.698		1.747		1.393	
*p*	0.0000		0.0000		0.0000		0.0565		0.0475		0.1600	
*No. of firms clusters*	92		92		84		95		95		87	

Regressions are estimated using OLS with standard errors clustered by company. All models include 2-digit SIC and year dummies.

* p < 0.1;

** *p < 0.05. In bold statistically significant coefficients of variables of interest*.

The economic effect of female directors on the nomination and remuneration committee is not negligible. The coefficient in column 3 of Table 4 indicates that an increase of one standard deviation in the proportion of female directors on the nomination and remuneration committee is associated with a 13% reduction in the growth rate of CEO compensation. The coefficient in column 3 of Table 5 indicates that having at least one female director on the remuneration and nomination committee is associated with a 23% reduction in the growth rate of CEO pay.

The models predicting the proportion of equity and option-based CEO pay displayed in columns 4–6 of Tables 4, 5 show positive coefficients for all the proxies of female board and committee membership. However, none of these coefficients are statistically significant at conventional levels. Altogether, our results suggest that female directors on the remuneration committee are associated to lower growth rates of CEO pay, no specific effect appears to exist in terms of the use of long term incentives in the executive pay mix.

In terms of types of directors, Tables 6, 7 suggest that the proportion of proprietary directors is associated to lower levels of CEO pay growth. The coefficient in the second column of Table 6 indicates that an increase of one standard deviation of the proportion of proprietary female directors is associated with a 10% decrease in executive pay growth. The second model in Table 7 indicates that having female proprietary directors is associated with a 26% lower growth rate in CEO pay.

**Table 6:** Regression results of CEO pay on executive, proprietary and independent female directors' weighting.

	**Relative annual increase of CEO pay**						**Proportion of equity and option based CEO pay**					
	**Model 1**		**Model 2**		**Model 3**		**Model 4**		**Model 5**		**Model 6**	
	**B**	**t**	**B**	**t**	**B**	**t**	**B**	**t**	**B**	**t**	**B**	**t**
Proportion of independent board female directors	0.483	0.72					0.393	1.58				
Proportion of proprietary board female directors			−1.401^*^	−**1.78**					−0.117	−0.53		
Proportion of executive board female directors					2.673	1.01					−0.459^**^	−**2.38**
Proportion of board independent directors	0.148	0.64	0.0814	0.42	0.281	1.34	−0.244^*^	−1.96	−0.181	−1.63	−0.173	−1.63
Logarithm of board size	0.116	1.05	0.134	1.23	0.120	1.06	0.0266	1.07	0.0285	1.10	0.0263	1.01
Firm logarithm of total assets	−0.0128	−0.45	−0.0146	−0.52	−0.0142	−0.50	0.000735	0.13	0.000346	0.06	0.000837	0.14
Return on assets	−0.323	−0.86	−0.350	−0.93	−0.259	−0.86	−0.00681	−0.08	−0.0123	−0.15	−0.0231	−0.27
Debt to assets ratio	−0.0868	−0.59	−0.100	−0.66	−0.0815	−0.61	0.0144	0.50	0.0114	0.45	0.0111	0.44
Annual market return	0.0453	0.45	0.0549	0.58	0.0695	0.72	−0.0616^**^	−2.21	−0.0511^*^	−1.87	−0.0531^*^	−1.94
Book to market ratio	0.0146	1.49	0.0160^*^	1.67	0.0143	1.43	−0.000780	−0.35	−0.000525	−0.25	−0.000599	−0.26
Constant	0.0993	0.22	0.227	0.50	0.0664	0.15	−0.0378	−0.34	−0.0251	−0.21	−0.0318	−0.28
*No. of Obs*.	243		236		213		297		290		264	
*Adjusted R^2^*	0.0584		0.0720		0.0545		0.184		0.178		0.157	
*F*	4.862		4.492		3.818		1.698		1.747		1.393	
*p*	0.0000		0.0000		0.0000		0.0565		0.0475		0.1600	
*No. of firms clusters*	92		92		84		95		95		87	

Regressions are estimated using OLS with standard errors clustered by company. All models include 2-digit SIC and year dummies.

* p < 0.1;

** *p < 0.05. In bold statistically significant coefficients of variables of interest*.

**Table 7:** Regression results of CEO pay on executive, proprietary and independent female directors' presence.

**Variable**	**Relative annual increase of CEO pay**						**Proportion of equity and option based CEO pay**					
	**Model 1**		**Model 2**		**Model 3**		**Model 4**		**Model 5**		**Model 6**	
	**B**	**t**	**B**	**t**	**B**	**t**	**B**	**t**	**B**	**t**	**B**	**t**
Independent female directors presence at the board	0.130	0.86					0.0313	0.62				
Proprietary female directors presence at the board			−0.267^**^	−**2.32**					0.0512	0.99		
Executive female directors presence at the board					0.339	1.04					−0.0634^**^	−**2.35**
Proportion of board independent directors	0.122	0.58	0.0319	0.15	0.274	1.30	−0.197	−1.60	−0.127	−1.25	−0.172	−1.62
Logarithm of board size	0.0994	0.84	0.166	1.49	0.116	1.05	0.0232	0.92	0.0174	0.75	0.0269	1.03
Logarithm of total assets	−0.0112	−0.39	−0.0165	−0.60	−0.0151	−0.53	0.00109	0.18	0.00121	0.20	0.00102	0.18
Return on assets	−0.315	−0.82	−0.362	−0.97	−0.261	−0.86	−0.0104	−0.12	−0.00781	−0.09	−0.0240	−0.28
Debt to assets ratio	−0.0767	−0.50	−0.110	−0.73	−0.0828	−0.61	0.0147	0.53	0.0150	0.60	0.0112	0.44
Annual market return	0.0417	0.42	0.0496	0.54	0.0686	0.71	−0.0554^*^	−1.93	−0.0502^*^	−1.93	−0.0532^*^	−1.94
Book to market ratio	0.0145	1.51	0.0159^*^	1.69	0.0143	1.43	−0.000691	−0.31	−0.000976	−0.43	−0.000604	−0.26
Constant	0.0948	0.21	0.245	0.54	0.0955	0.22	−0.0394	−0.34	−0.0613	−0.46	−0.0360	−0.31
*No. of Obs*.	243		243		243		297		297		297	
*Adjusted R^2^*	0.0618		0.0741		0.0647		0.181		0.184		0.181	
*F*	5.005		4.602		4.549		1.847		1.914		1.775	
*p*	0.0000		0.0000		0.0000		0.0330		0.0258		0.0430	
*No. of firms clusters*	92		92		92		95		95		95	

Regressions are estimated using OLS with standard errors clustered by company. All models include 2-digit SIC and year dummies.

* p < 0.1;

** *p < 0.05. In bold statistically significant coefficients of variables of interest*.

We also display in the sixth column of Table 6 a significant and negative relationship between the proportion of board executive female directors and the proportion of equity and option-based CEO remuneration. Nevertheless, the economic importance of this effect is relatively small. An increase in one standard deviation in the proportion of executive female directors is associated with a 0.19% reduction in the proportion of equity and option-based remuneration. The corresponding model in Table 7 indicates that having one or more female executive directors is associated with a 6% lower proportion of equity and option- based CEO remuneration.

Table 8 shows the results for the analysis of the relationship between female directorships and the proportion of shareholders' opposition to the managerial compensation plan voted at the annual general meeting (dissent say on pay vote). Coefficients for all the proxies of female directorships are negative, but only statistically significant in the case of female directorships on the remuneration and nomination committee. The coefficient in the third column of Table 8 indicates that a one standard deviation in the proportion of female directors on the remuneration and nomination committee is associated with a lower 0.8% dissent say on pay vote. This reduction in say on pay opposition might appear to be negligible but represents 30% of the average negative say on pay vote. This result is consistent with the moderating role of female directors on this committee reported in Tables 4, 5.

**Table 8:** Regression results of Dissent say on pay vote on female directors' weight.

**Variable**	**Proportion of dissent say on may vote**					
	**Model 1**		**Model 2**		**Model 3**	
	**B**	**t**	**B**	**t**	**B**	**t**
Proportion of board female directors	−0.0346	−1.00				
Proportion of audit committee female directors			−0.000724	−0.03		
Proportion of remuneration committee female directors					−0.0478^***^	−**2.73**
Proportion of board independent directors	−0.0106	−0.42	−0.0108	−0.42	−0.00388	−0.16
Logarithm of board size	0.0124*	1.79	0.0123	1.52	0.0127*	1.80
Logarithm of total assets	0.00267	1.61	0.00254	1.43	0.00233	1.35
Return on assets	0.0371	1.42	0.0386	1.52	0.0429	1.61
Debt to assets ratio	0.00709	0.75	0.00685	0.71	0.0102	0.83
Annual market return	0.00547	0.87	0.00441	0.71	0.00769	1.02
Book to market ratio	−0.000684	−0.84	−0.000659	−0.80	−0.000364	−0.49
Constant	−0.0214	−0.94	−0.0247	−1.06	−0.0288	−1.27
*No. of Obs*.	371		361		329	
*Adjusted R^2^*	0.0537		0.0504		0.0617	
*F*	4.149		4.212		4.102	
*P*	0.0000		0.0000		0.0000	
*No. Clusters*	108		108		106	

Regressions are estimated using OLS with standard errors clustered by company. All models include 2-digit SIC and year dummies.

* p < 0.1;

*** *p < 0.01. In bold statistically significant coefficients of variables of interest*.

Moreover, with the set of control variables used across all the estimated models we have obtained evidence of a negative relationship between the firm's market return and the proportion of equity and option-based CEO pay. This result could be explained as an attempt of low performing firms to improve their market returns by providing variable incentives to their CEOs. Option and equity-based CEO pay formulas provide the CEOs with incentives to create value for shareholders, that is, to align their interests with those of the firm.

Overall, our results suggest that female directors on the nomination and remuneration committee are both associated with lower levels of CEO pay growth and lower levels of shareholder opposition to the executive remuneration plan. This result suggests that the moderating role of female remuneration committee members is valued positively by firm owners. All in all, these results provide support to our hypothesis that female directors contribute toward CEO pay moderation. We also have partial support for the hypothesis that female directors reduce the use of option-based executive compensation given that our results only hold for executive female directors. This can be explained by the natural risk aversion of executives and at the same time by the low proportion of option-based executive pay in the Spanish market which renders this matter less relevant when designing executive pay plans.

## Discussion

The current debate about women on corporate boards of directors focuses on closing the pay gap and opening company boardrooms up to more women. The search for a political and social solution has led to additional and new legislation that involves an increased use of measures for the advancement of women to reduce gender gaps.

In addressing gender equality, we need to recognize that in all developed countries there are concerns about how to improve the situation of women in different settings of the professional arena. Women have reached higher education levels than men, so at the European level women represent 60% of all workers with higher education but only a 45% of total employment. However, when we look at the management positions, there seems to be a “glass ceiling” that prevents women from accessing positions of power and greater responsibility. Our sample shows that the percentage of female executive directors is low with only 5% of listed firms having female executive directors.

In Spain, following the implementation of the Organic Law 3/2007 on effective equality between men and women, it was recommended, not imposed, to increase and to include a minimum 40% of women on the boards of directors. The progressive implementation of effective gender equality legislation has favored the increase of women on the boards of directors in Spain. Their presence has gone from 6% in 2007 up to 17% nowadays. A very limited advance, more so when we consider that only 5% of executive directors are women. This means that most women are proprietary or independent directors with less decision-making power than executive directors on the board of directors. Nonetheless, there is a gap between the moment when new legislation is passed and the moment on which the implementation of such measures takes place (for instance, García-Izquierdo et al., 2010, 2015), so a progressive incorporation of women to boards during coming years is expected despite the objective of such 40% should be obtained by 2015 (eight years after legislation has passed). Nonetheless, reasons for this scarce implementation are not clear. We can say that 3/2007 Law explicitly state a recommendation (i.e., try to reach) but not a compulsory objective, nor any punishment nor any reward. Probably, in this matter of gender in traditional organizational contexts “hard law” would be more effective than “soft law.” Simultaneously, as Boards are mainly composed by men probably they are aware or not committed enough for such social objective. Moreover, the economic crisis could easily have an effect on shaping organizational decisions to more tangible measures.

All in all, this paper corroborates that although the legislation in Spain presents a margin for improvement, as soft law, the incorporation of women appears to exert a positive effect in terms of higher wage moderation, and restraint in the use of long-term variable remuneration systems.

We have shown also how the presence of female directors on the board of directors and on the remuneration committee can provide a valuable tool for moderating CEO pay. Moreover, our results show that the presence of female directors on the remuneration committee is associated with lower growth rates of CEO pay, which can be considered a better organizational outcome in terms of good governance. This represent a clear proxy evidence of minority influence on boards, despite it is quite difficult to reveal the underlying mechanisms that are taking place in boards decision-making processes as is well known the meetings are more like a blackbox.

This research has been focused on the analysis of the relationship between the presence of women and CEO remuneration policy on boards of directors, but it would be interesting to include in the analysis other personal characteristics of the directors such as previous professional experience on other boards and academic and professional background. Nevertheless, we have seen that this limitation should not be particularly crucial as we can expect that personal, educational and professional profiles of male and female directors of companies operating in Spain do not differ significantly, even in family businesses due the implementation of the protocol.

Moreover, it should be quite interesting and appealing to unveil group processes and decision making mechanisms in order to be more informative and transparent for the stakeholders.

## Author contributions

All authors listed have made a substantial, direct and intellectual contribution to the work, and approved it for publication.

### Conflict of interest statement

The authors declare that the research was conducted in the absence of any commercial or financial relationships that could be construed as a potential conflict of interest.
